# Treatment of Nystagmus in Brainstem Cavernous Malformation with Botulinum Toxin

**DOI:** 10.7759/cureus.553

**Published:** 2016-04-01

**Authors:** Yi-Ren Chen, Douglas Fredrick, Gary K Steinberg, Yaping J Liao

**Affiliations:** 1 Department of Neurosurgery, Stanford University Medical Center; 2 Ophthalmology, Stanford University Medical Center

**Keywords:** nystagmus, oscillopsia, botulinum toxin, cavernous malformation, vascular malformation

## Abstract

We report a long-term eye movement study of a 68-year-old female with pontomedullary junction cavernous malformation whose dysconjugate nystagmus was treated with retrobulbar botulinum toxin A injections. Sequential, bilateral retrobulbar injections of botulinum toxin A were performed. Injections immediately decreased oscillopsia and nystagmus, and improved visual acuities. One to three months following injection, three-dimensional infrared oculography measured a significant 39-100% (P = 0.001) decrease in nystagmus amplitudes at multiple dimensions. This improvement diminished by six months in the right eye but sustained for about one year in the left eye. Over two years, botulinum toxin A injections were performed twice in the left eye and five times in the right eye. Our study supported the safe and effective use of repetitive retrobulbar botulinum toxin A injections in symptomatic nystagmus that failed medical therapy.

## Introduction

Cavernous malformations are irregular, honeycomb-shaped blood-filled lesions composed of thin-walled vascular spaces without intervening brain parenchyma. They have an estimated prevalence of 0.5% of the population [1-2] and represent 5–13% of all intracranial and spinal vascular malformations [3-4]. Common presenting symptoms of cerebral cavernous malformations include headache, seizure, subarachnoid or intraparenchymal hemorrhage, altered mental status, and hydrocephalus. Cavernous malformations in the brainstem are particularly debilitating due to impaired ocular motor control, which leads to oscillopsia, binocular diplopia, dizziness, and nystagmus [5-6].

While botulinum toxin A has been used to treat acquired and congenital nystagmus [7-12], it has not been reported to be effective on eye movement abnormalities beyond one to three months [7-9, 13, 14]. There is one report of its use to help three patients with pontine hemorrhage [14]. We present here the first one-year eye movement study to investigate the effects of retrobulbar botulinum toxin A injections to treat nystagmus associated with brainstem cavernous malformation. Informed consent was obtained from the patient for this study.

## Case presentation

### Patient

**Figure 1:**
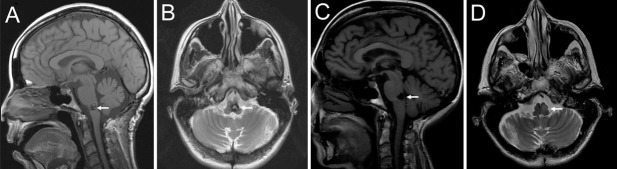
Brain magnetic resonance images of the pontomedullary junction cavernous malformation before and after resection. Preoperative sagittal T_1_- (A) and axial T_2_-weighted (B) images show heterogenous signal at the dorsal medulla (arrows) characteristic of a cavernous malformation with prior hemorrhage. Postoperative sagittal T_1_- (C) and axial T_2_-weighted (D) images show resection of the cavernous malformation and well-healed surgical site at the dorsal medulla (arrows).

A 68-year-old female presented with multiple brainstem hemorrhages from a 9 x 10 x 12 mm pontomedullary junction cavernous malformation (Figure 1) resulting in recurrent left, then bilateral, cranial nerve VI palsy, right-sided hemiparesis with hyperreflexia, bilateral decreased sensation, and gait instability.

The patient underwent left suboccipital craniotomy with microscopic computer-assisted resection of the lesion under mild hypothermia using intraoperative electrophysiological monitoring and functional brainstem mapping. Immediately postoperatively, the patient exhibited near complete bilateral horizontal gaze palsy consistent with bilateral cranial nerve VI palsy, bilateral internuclear ophthalmoplegia, esotropia, left hypertropia, bilateral nystagmus, right facial numbness, left worse than right facial weakness, left hearing loss, transient swallow difficulties, right hemiparesis, and right-sided ataxia.

Her nystagmus was noted immediately post-op and resulted in persistent oscillopsia. Her oscillopsia was treated with multiple medical therapies, none of which significantly improved her symptoms. These included gabapentin (2700 mg daily), memantine (10 mg daily), pregabalin (300 mg daily), scopolamine patch (1.5 mg daily), lorazepam (1.5 mg daily), and baclofen (10 mg three times daily).

For her binocular diplopia, she was initially treated with Fresnel prism immediately after craniotomy. Two years after craniotomy, the patient underwent right medial rectus recession and transposition of the vertical rectus muscles temporally in the left eye. This led to resolution of her hypertropia but persistent exotropia. Four years after craniotomy, she had bilateral minimal horizontal eye movement, full vertical duction, and a 15 prism diopter exotropia.

For her cranial nerve VII paresis, lagophthalmos, and severe exposure keratopathy in the left eye, the patient had gold weights implanted in the left superior eyelid. She subsequently underwent left hypoglossal to facial nerve anastomosis, which allowed greater left facial nerve function, leading to the later removal of the gold weights.

### Eye movement recording and statistical analysis

To assess her ocular oscillations and monitor the response to treatment, we performed 60-Hz binocular three-dimensional infrared oculography (3D-VOG, SensoMotoric Instruments, Teltow, Germany) over one and half years. We analyzed the data using custom-written programs in IgorPro and Matlab and determined statistical significance with Wilcoxon signed-rank test.

### Results 

Given the failure of medical therapy, we performed sequential retrobulbar injections of 25-30 units of botulinum toxin A as treatment for continuous oscillopsia. We used a 25-gauge blunt retrobulbar needle, which minimized the risk of ocular perforation and increased the likelihood that the botulinum toxin would weaken all four recti muscles. The needle was inserted at the inferolateral border of the orbit and directed straight back and then medially and superiorly toward the orbital apex. Following negative aspiration of blood, the botulinum toxin was injected, and then the needle was withdrawn. After each botulinum toxin A injection, the patient experienced no change in duction or ocular alignment, although she had transient ptosis with some injections.

**Figure 2:**
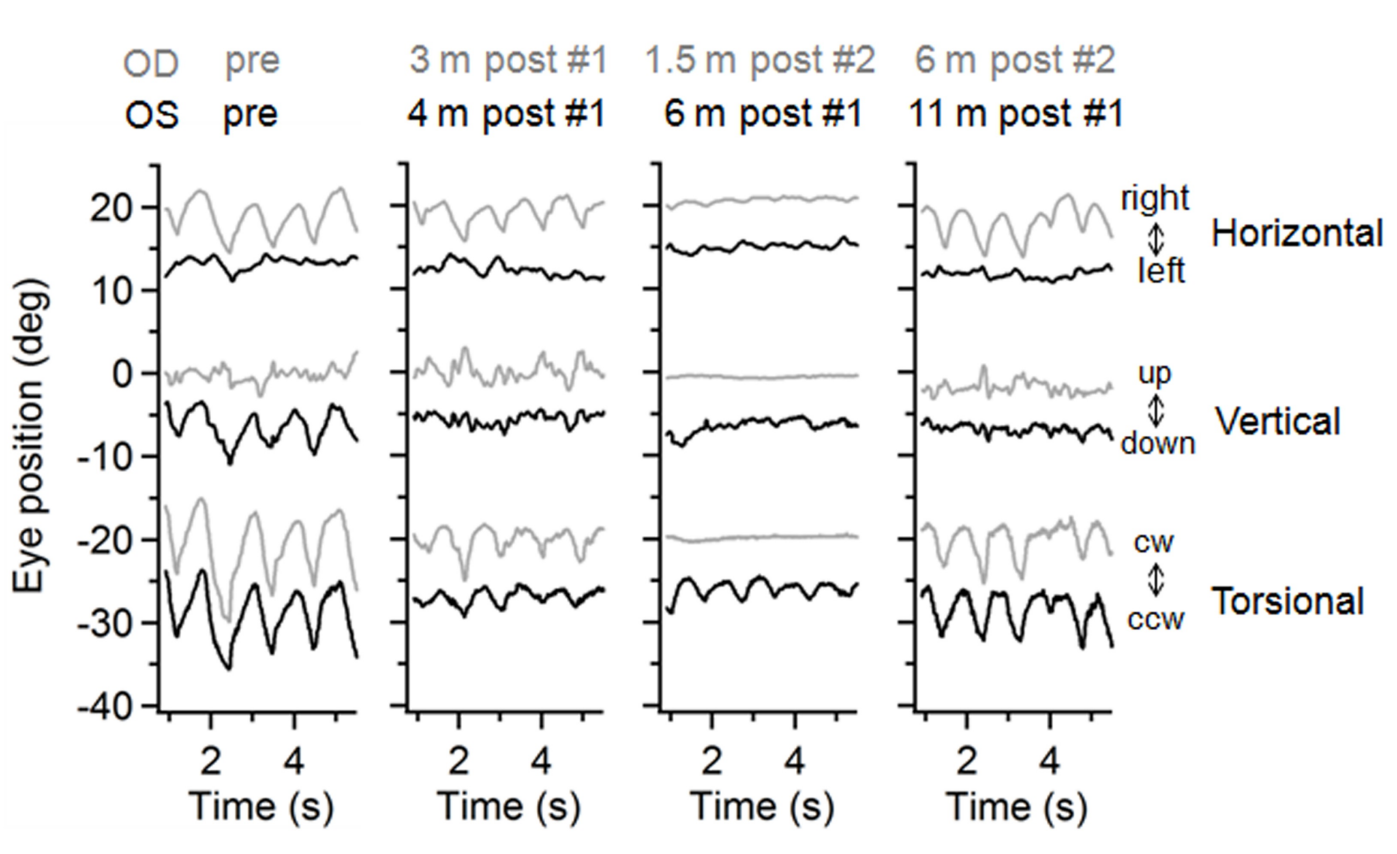
Retrobulbar botulinum toxin A injection significantly improved nystagmus amplitudes over months. Three-dimensional eye movement waveforms before and after injections. Before treatment, the patient had 1-Hz dysconjugate nystagmus on 60-Hz infrared oculography, with the right eye (OD, gray traces) showing horizontal and torsional oscillations and the left eye (OS, black traces) exhibiting vertical and torsional oscillations.

We performed infrared oculography before and after retrobulbar botulinum toxin A treatment. Pre-treatment, there were 1-Hz oscillations that were conjugate in the torsional plane (right eye: 10.0 ± 1.0 deg, left eye: 8.4 ± 0.6 deg) but dysconjugate in the other planes (Figure 2).

The right eye exhibited horizontal oscillations of 5.7 ± 0.5 deg and relatively small vertical oscillations (2.0 ± 0.3 deg), while the left eye had 6.0 ± 0.6 deg oscillations in the vertical plane and little horizontal oscillations (1.8 ± 0.3 deg).

Within two weeks following each injection, the patient reported symptomatic improvement in visual function and oscillopsia. Her visual acuities improved to 20/40 right eye and 20/50 left eye. Eye movement recordings showed persistent dampening of nystagmus amplitudes in both eyes three to four months following one botulinum toxin injection (Figure 2). The injection was particularly efficacious in her left eye compared with the right. While the effect of botulinum toxin was not expected to last longer than three months, at six months following one injection, the patient’s left eye still had a 83% decrease in vertical nystagmus amplitude (P < 0.00001), which was sustained at 11 months (P < 0.00001) (Figure 2). In the torsional plane, there was also a 70% decrease in nystagmus amplitude (P < 0.00001) at six months. This improvement was still present at 11 months post-injection, although only at a 36% decrease (P = 0.0002). The relatively sustained amelioration in the left eye meant the patient did not require repeat botulinum toxin injection in that eye until about two years later.

**Figure 3:**
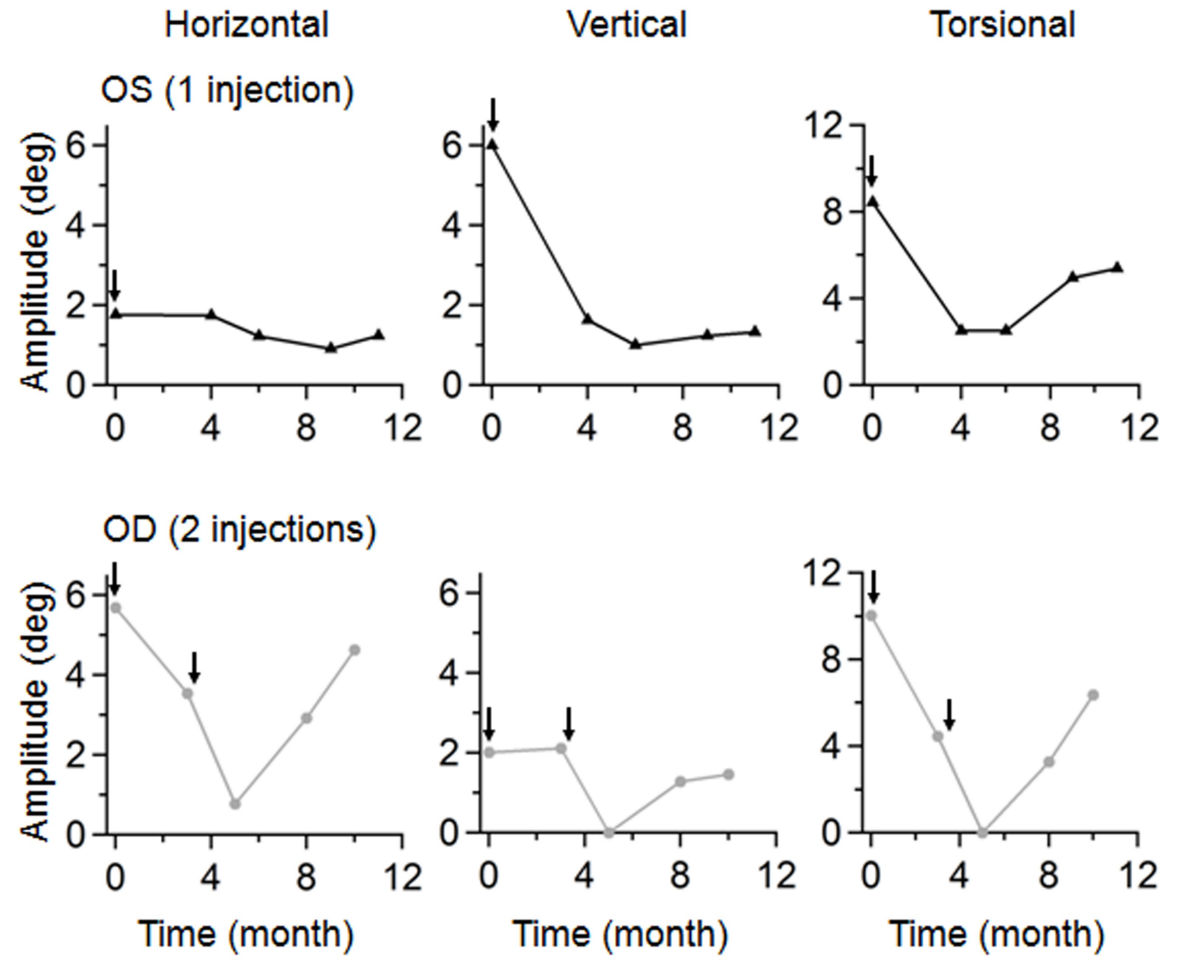
Graph of nystagmus amplitude over time. Following one injection (arrow), the left eye (OS, top) exhibited sustained benefit over months, while the right eye (OD, bottom), which received two injections (arrows), demonstrated improved nystagmus at 1.5 months after the second injection and then deteriorated gradually over the following 6 months.

In the right eye, one injection led to a 65% decrease in the horizontal (P < 0.00001) and a 39% decrease in the torsional amplitudes (P < 0.00001). Despite these improvements, symptomatic oscillopsia in the right eye led to repeat injection at three months after first injection. One-and-half months after the second injection, there was an 86% decrease in the nystagmus amplitude in the horizontal plane (P < 0.00001) and a 100% decrease in the torsional plane dimension (P < 0.00001) compared to pre-injection baseline (Figure 2). Unfortunately, these benefits were not sustained. At six months after the second injection or 10 months after the initial injection, the amplitudes of the nystagmus reverted to near pre-injection values (horizontal 18% decrease compared to pre-injection, P = 0.07; torsional 37% decrease compared to pre-injection, P = 0.0002) (Figure 3).

Because of the relative lack of sustained effects, the right eye was injected five times over two years, with excellent results each time. As expected for retrobulbar injections to treat a brainstem problem, there was no change in the frequency or velocity waveforms of the recurrent nystagmus compared to the baseline in both eyes.

## Discussion

We report a long-term clinical and quantitative eye movement study of the efficacy of retrobulbar botulinum toxin A injection to treat dysconjugate nystagmus in a 68-year-old patient who was status post left suboccipital craniotomy for treatment of pontomedullary junction cavernous malformation and failed conservative treatment. One retrobulbar botulinum toxin A injection improved oscillopsia, visual acuity, and nystagmus amplitude for months in both eyes, with sustained effects longer than six months in the left eye and less than six months in the right eye. One injection to the left eye was efficacious for two years until another injection was needed, while the right eye required five injections in the same two-year period. 

By serially recording over one year and following clinically over three years, we showed that retrobulbar botulinum toxin injections have sustained efficacy of months to longer than one year. This is much longer than the previously reported one-to-six-month eye movement studies [9, 13-14] and clinical assessments [7-8, 10-11]. The mechanism of this sustained benefit is unknown since the effects of retrobulbar botulinum toxin have not been well studied in animal models, although the loss of efficacy is known to depend on nerve sprouting and formation of new neuromuscular junctions [7], and intramuscular injection into extraocular muscles in animals leads to increased number of neuromuscular junctions and myofiber remodeling within weeks [15-16].

Injection of botulinum toxin directly into the extraocular muscle has been used to treat nystagmus [7, 9, 11, 17-19], and eye movement recording has shown improved nystagmus amplitude at four weeks [9]. However, the retrobulbar route of botulinum toxin administration has advantages over direct injection of the medial and lateral rectus muscles, with less injury to the muscle, lower cost, and no need for electromyography [14]. In addition, the retrobulbar technique is easily performed and requires only one injection to affect multiple muscles. Although botulinum toxin has been proposed as treatment for nystagmus for about 20 years, it has not been widely used. Because retrobulbar botulinum toxin treatment involves repeat injections around the eye, this invasive treatment is often not considered by physicians or patients. Common side effects of retrobulbar botulinum toxin injection include ptosis and diplopia, from spreading of the toxin to nearby structures [7-9, 12-14].

## Conclusions

The efficacy of botulinum toxin treatment can be unpredictable and typically lasts about three months, although in our patient we found sustained efficacy for longer than one year in one eye with repetitive injections. Since there are relatively limited options for treatment of nystagmus, our data provide support for treatment consideration of retrobulbar botulinum toxin A injection, since treatment can be performed safely with significant benefit to the patient’s visual experience and function.
